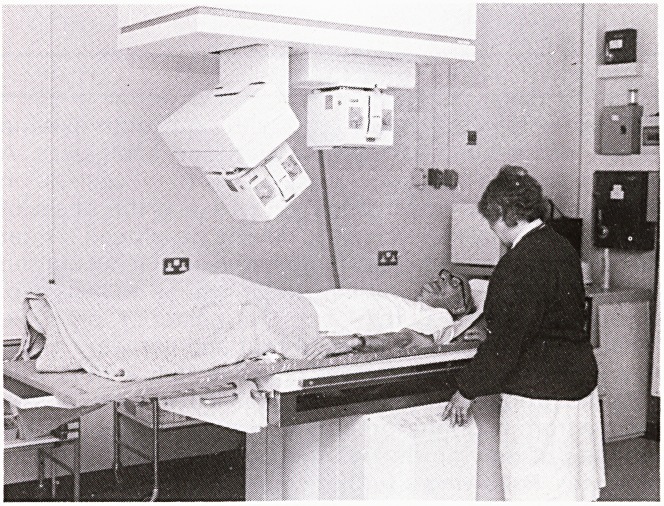# From Our Correspondents

**Published:** 1988-08

**Authors:** 


### From our correspondents

### CONCRETE CANCER AT THE ROYAL DEVON AND EXETER.

Dismay, despondency and delight—all three emotions were experienced when rumour gave way to fact about 'concrete cancer' affecting the Royal Devon & Exeter Hospital. Dismay: would it fall down? Better knowledge about modern steel construction techniques soon allayed these fears. Careful monitoring from the start has shown the Tower of Pisa is infinitely more likely to fall first.

Despondency soon followed: here was a modern hospital likely to be unserviceable within five years. In some ways emergency evacuation might have been less sapping to morale but the priceless national virtue of resilience in the face of adversity came to the rescue. We soon realised we had an opportunity to rebuild on a scale and within a time virtually unknown in the NHS; this could only be achieved by local planning. Within a short time Mr David King, District General Manager, skilfully assembled a working group comprising medical, managerial and works staff who have worked quickly, cordially and cohesively on this mammoth task.

Establishing a diagnosis is just as important in treating a hospital as in treating a human. Like many patients early manifestations of disease were ignored but the cracks and crumbling came to a point where specialist advice was needed. Once the diagnosis had been made a second opinion was sought and 'biopsies' carried out. The diagnosis was clear—the prognosis less clear. Nonetheless it looked unlikely that even the least affected parts would last longer than 10 years and the tower block no more than five. The decision seemed clear locally. It was fortunate the SWRHA subcommittee was chaired by a surgeon skilled in diagnosing and treating cancer—they too quickly came to the same conclusion.

Coming at a time when the health service was going through one of its now so familiar phases of self-examination we had an opportunity to think what might be needed in the 21st century—in spite of flatulent political rodomontade in the lead up to the last general election, this was not an opportunity given to most Health Authorities! Time and effort was spent talking to every group in the hospital—clinical, laboratory, nursing, administrative and ancillary staff. Some clinical members of the working group learned a lot in the process. The most important decision, and the one that initially caused the greatest soul-searching, was whether to go for a 'nucleus' hospital or to have it wholly designed afresh. In retrospect we could have saved a lot of time if the Department of Health had made it clear from the outset that the money for a replacement would only stretch to a nucleus hospital.

Much has been written about 'nucleus' designs. It would be the apotheosis of dottiness for each new development to spend money on design features common to all hospitals. Nucleus design means this basic work has been done, but there is still considerable flexibility. It is rather like children's building bricks—there are some constraints but still plenty of room for creativity. Once this was mastered we moved forward with speed. Joined at this stage by architects and planners who must often have found our sometimes crazy ideas exasperating the clinicians learned a lot about hospital construction (and 'planning' and 'fire regulations'!).

In record time we have got to the stage where preliminary works are in progress and the contract for the first priority—rebuilding the ward tower block—is out to tender. One building is being demolished which gives the structural engineers a chance to examine the 'pathology'; we now have a chance of 'histological confirmation' of the diagnosis. Our task is not yet complete as now we have to continue with planning the second priority of replacing theatres, radiology and out-patient departments as well as special departments like cardiology, pulmonary function, clinical measurements, remedial services, and radiotherapy. Included in this second priority will be catering, medical records and all those unsung departments without which a hospital would not run.

Dismay? Despondency? Yes both emotions at differing times but for most of us intimately involved in planning the replacement a sense of delight in achieving something few people have the chance to do in their lifetime namely to plan and see a complete new hospital constructed.

BRIAN KIRBY.

### THE LITHOTRIPTOR AT SOUTHMEAD HOSPITAL: A DONATION FROM THE DAWN JAMES CHARITABLE TRUST

The management of stones in the upper urinary tract has changed dramatically during the past 10 years. The development of percutaneous renal surgery and then the introduction of Extracorporeal Shock Wave Lithotripsy (ESWL) have made open surgery for stones almost obsolete. ESWL has been pioneered by the West German manufacturers, Dornier, who produced the original lithotriptor in 1980 after many years research. Treatment on their first machine involved placing the patient in a water bath on a hydraulic hoist and the pain from the shock waves necessitated general or regional anaesthesia. Progress in the development of these machines has been rapid and a variety of lithotriptors are now available. As a result of the generous donation from the Dawn James Charitable Foundation, a Siemens Lithostar has recently been installed at Southmead Hospital. This is a second generation machine providing a versatile system for a fully integrated stone service. The Unit was officially opened by Mr John James CBE LLD on 12 July 1988.

A lithotriptor shatters a stone by generating a shock wave that can be focussed on to the renal or ureteric stone. In fact little is known about the mechanism of stone disintegration and the lithotriptors in popular use at the present time generate shock waves in different ways. Localisation of the stone is performed by ultrasonic or radiological means; whilst ultrasound identifies radio-lucent stones, small radio opaque stones in the ureter are more readily identified on an x-ray screen.

The Lithostar localises the stone by means of biplanar x-ray sources. There are two shock heads, one for each side of the urinary tract, that are capable of producing 90–120 shocks per minute. The patient lies on a table that can be used as a uroradiological table for screening purposes and thus cystoscopies, insertion of stents, ureterorenoscopy (URS) and even percutaneous nephrolithotomy (PCNL) can be performed in the Unit.

The initial experience of the Lithostar has been most encouraging. 80% of the patients are treated without general or regional anaesthesia but they are given mild sedation using Temazepam and Diclofenac. This has meant that the majority attend as a day case. Stones up to 1.5 cm in diameter, situated in the kidneys or in the upper and lower thirds of the ureter are readily amenable to treatment in this way. Larger stones, 2 cm or more in diameter, produce multiple fragments when disintegrated and these can be held up in the ureter forming what is termed a Steinstrase or stone street. To avoid this a double J-stent is normally introduced before commencing treatment and this is performed under a general anaesthetic. In patients with a large staghorn calculus, a percutaneous debulking procedure can initially be performed to reduce the size of the stone and then the residual fragments are disintegrated by ESWL. There does appear to be a wide variation in the number of shocks required to shatter a stone. Some stones have been fragmented with less than 500 shocks whereas others have required many thousands delivered over a number of sessions. The composition of the stones probably accounts for this and cystine stones are notoriously hard and may not disintegrate with ESWL.

Careful follow up is essential following ESWL because complications can be insiduous. Some patients are travelling long distances for treatment and good communication between the Unit and the referring consultants is essential. Pain in the loin or pyrexia require urgent investigation as drainage to the kidney may be necessary. Obstruction to a kidney by a Steinstrasse, particularly in cases of infected stones, can give rise to septicaemia. Patients are discharged following treatment on Trimethoprim and a plain x-ray of the urinary tract is performed one week after treatment.

Assessment of the first 50 patients treated on the lithotriptor showed that 30 were stone free after one month. 18 out of 29 calyceal stones, 4 of 9 pelvic stones, and 8 of 12 ureteric stones had been completely disintegrated. The treatment or urinary tract stones has clearly entered a new era.

R. C. L. FENELEY

**MOTORS, MEDICINE AND GILBERT LANG**

Life has its sadnesses. Few are more painful than the passing of respected colleagues.

Pathologists are a poor breed when it comes to treating patients. Most of our clinical colleagues forgive this failing, and know that we should never be allowed to become first-aid people. Nonetheless, they understand that we are keen observers of the diagnostic process. Some of our other colleagues in the laboratories are more practical. Not many people on the outside will know how much we owe to our mortuary staff.

Recently I was involved in an impressive non-medical diagnosis.

Alas, the occasion involved an unsuccessful attempt to attend the funeral of a very much-respected mortuary attendant at the Royal Infirmary, the late Gilbert Lang. Unfortunately, I never made it. Despite the black tie and sober suiting my car decided that the hill up from the BRI was too much. The rev counter spun round to impossible counts, and the entire machinery jammed up. An innocent thought at first was that over revving, emotion or flooding of the engine were the cause. Time passed on the road side and still nothing happened.

So, what to do? Call in the RAC. OK, you are then a proper patient. Take it easy and watch. There is much to learn. First there was the comforting reassurance. No, it isn't just a simple flooding. Ten minutes later one learns that the likely problem is a failure of the control box. A what? "You'll need to go to a garage," said the RAC man. Fine, so let's do just that. An efficient tail-of-the van hook up followed.

Now we enter the diagnostic set up in the real world. We arrived at the garage. "This time it's an entry in real style." I said feebly to my favourite garage man, feeling embarrassed about the bright blue and red RAC towing van with its flashing yellow lights. "Why hello, how nice to see you," said the favourite garage mechanic.

Well, at this point the two of them—mechanic and RAC man—fell into deep professional conversation about how much the RAC tow van would cost on the second-hand market and where they were available for auction. It was rather similar to conversations about reconditioned endoscopes, electron microscopes or reduced-price monoclonal antibodies.

The next thing to happen, just as the helpful RAC man was on the point of going off, was a quiet question from my garage man. "Did he look at this?" No, even I—with very little knowledge of under-the-bonnet happenings—knew that the fuse box had not been checked out.

Oh ho. It's all rather like our own field. I'm glad to have a master diagnostician on my side before the treatment starts. I don't know how much it would have cost to have gone elsewhere. Anyway the fun of watching experts politely disagree is worth the time. It just makes you feel so much more at home.

Gilbert Lang. I salute you. You would have enjoyed the whole thing. Apologies for not making it.

J. D. DAVIES

**CROHN'S DISEASE – A MISNOMER**

Referring to Michael Harmer's article, Feb 1988, p 9. The Editor has permission to reveal this congratulatory message.

To Michael Harmer from Michael Reilly–

Dear Michael,
B. B. Crohn
Is not now alone
In describing what a blight is
Ileitis,
And a notable feat is
Debunking eelieetis.
Yours
Michael.

MICHAEL REILLY MS,
Yelverton,
Devon.

## Figures and Tables

**Figure f1:**